# Reevaluation of Pluripotent Cytokine TGF-β3 in Immunity

**DOI:** 10.3390/ijms19082261

**Published:** 2018-08-01

**Authors:** Toshihiko Komai, Tomohisa Okamura, Mariko Inoue, Kazuhiko Yamamoto, Keishi Fujio

**Affiliations:** 1 Department of Allergy and Rheumatology, Graduate School of Medicine, The University of Tokyo, Tokyo 113-8655, Japan; tkomai-hok@umin.ac.jp (T.K.); mariinoue-tky@umin.ac.jp (M.I.); kazuhiko.yamamoto@riken.jp (K.Y.); 2 Department of Functional Genomics and Immunological Diseases, Graduate School of Medicine, The University of Tokyo, Tokyo 113-8655, Japan; 3 Max Planck-The University of Tokyo Center for Integrative Inflammology, The University of Tokyo, Tokyo 153-8505, Japan; 4 Laboratory for Autoimmune Diseases, Center for Integrative Medical Sciences, RIKEN, Kanagawa 230-0045, Japan

**Keywords:** transforming growth factor-β3, transforming growth factor-β1, immune tolerance, regulatory T cell, autoimmune disease, immunometabolism, fibrosis

## Abstract

Transforming growth factor (TGF)-βs are pluripotent cytokines with stimulatory and inhibitory properties for multiple types of immune cells. Analyses of genetic knockouts of each isoform of TGF-β have revealed differing expression patterns and distinct roles for the three mammalian isoforms of TGF-β. Considerable effort has been focused on understanding the molecular mechanisms of TGF-β1-mediated immune regulation, given its pivotal role in prohibiting systemic autoimmune disease. In recent years, functional similarities and differences between the TGF-β isoforms have delineated their distinct roles in the development of immunopathology and immune tolerance, with increased recent attention being focused on TGF-β3. In addition to the characteristic properties of each TGF-β isoform, recent progress has identified determinants of context-dependent functionality, including various cellular targets, cytokine concentrations, tissue microenvironments, and cytokine synergy, which combine to shape the physiological and pathophysiological roles of the TGF-βs in immunity. Controlling TGF-β production and signaling is being tested as a novel therapeutic strategy in multiple clinical trials for several human diseases. This review highlights advances in the understanding of the cellular sources, activation processes, contextual determinants, and immunological roles of TGF-β3 with comparisons to other TGF-β isoforms.

## 1. Introduction

The transforming growth factor (TGF)-β superfamily comprises more than 40 members, including the TGF-βs, bone morphogenetic proteins (BMPs), growth and differentiation factors (GDFs), activins, and nodals [1,2,3]. TGF-β superfamily members share several biological functions, but also possess specific or opposite functions under some conditions [1].

The TGF-βs are involved in a variety of biological functions in cellular activities, fibrosis, and immune responses, in addition to their crucial roles in tissue homeostasis [1]. Three mammalian TGF-β isoforms (TGF-β1, TGF-β2, and TGF-β3) have been identified, with 70–82% amino acid homology [4]. TGF-β1 has been extensively investigated in immunity, because TGF-β1 is expressed predominantly by various immune cells [5], and deficiency of *Tgfb1* results in fatal systemic autoimmune disease [6,7]. TGF-β2, however, is thought to play an insignificant role in the immune system due to its low expression in immune cells [8]. Recent accumulating evidence on the expression and function of TGF-β3 in immunity has revealed similarities and differences between TGF-β1 and TGF-β3 [9,10]. In this review, we focus on the molecular characteristics, immunological roles, and future possibilities of TGF-β3 in comparison to TGF-β1.

## 2. TGF-β Superfamily Members and Immunity

TGF-β1, a prototypic TGF-β superfamily cytokine, was discovered in the early 1980s as an autocrine factor secreted by neoplastic cells to promote their transformation [11,12,13]. Mature TGF-β1 forms a disulphide-linked dimer of two identical chains derived from the C-terminus by proteolytic cleavage [14]. A number of proteins that share sequence similarity have been assigned to the TGF-β superfamily [14], and each TGF-β superfamily ligand signals by binding heteromeric complexes of transmembrane type I receptors and type II serine/threonine kinase receptors [1,15]. TGF-β superfamily members can be further subdivided into TGF-βs (TGF-β1, -β2, -β3), activins/inhibins/nodal proteins, and BMPs [15]. In this section, we discuss TGF-β superfamily members other than the TGF-βs and their impact on immunity.

Activins exist as either homo- or hetero-dimers of β-subunits, and the neonatal lethality with craniofacial abnormalities in activin-βA knockout mice indicates the importance of activin A during mammalian development [16]. Previous analyses demonstrate that activin A suppresses the generation and survival of B cells [17], cytokine and chemokine production by dendritic cells (DCs) [18], and the proliferation of thymocytes [19] and peripheral blood lymphocytes [20]. In T cells, activin A promotes TGF-β1-depdendent conversion of CD4^+^CD25^−^ T cells into forkhead box P3 (Foxp3)-expressing regulatory T cells (Tregs) [21], which play critical roles in immune homeostasis [22,23], in addition to inducing antigen-specific Tregs [24]. Although these findings suggest that activin A functions as a potent anti-inflammatory cytokine, activin A also has pro-inflammatory effects, and its systemic role in vivo has not been clearly demonstrated [25]. It has been reported that activin A is increased in synovial fluid of rheumatoid arthritis (RA) patients [26], and that activin A induces the proliferation of fibroblast-like synoviocytes [27]. Further studies are needed in order to characterize activin A activity in vivo and its influence on disease pathogenesis in humans.

BMPs were initially discovered as a factor that induces bone formation, and a number of crucial roles in embryogenesis, development, the skeletal system, and tissue homeostasis have since been identified and assigned to the BMPs. BMP2 and BMP4 knockout mice result in embryonic lethality, and BMP1, BMP7, and BMP11 knockout mice die shortly after birth [28]. BMPs are reported to regulate diverse immune cell types, such as DCs, macrophages, natural killer (NK) cells, B cells, and T cells [1]. BMPs generally enhance anti-inflammatory activities, and BMP2 and BMP4 support thymic organogenesis [1]. Since BMPs regulate neuronal and glial lineage cells as well as impacting immune responses, BMPs are potential therapeutic targets for the neurodegenerative autoimmune disease multiple sclerosis (MS) [29]. For example, injected neuronal precursor cells ameliorate the pathologies of experimental autoimmune encephalomyelitis (EAE), an animal model of MS, in a BMP4-dependent manner [30]. Furthermore, T cell secretion of BMP2, BMP4 and BMP5 is elevated in MS patients [31]. Although BMP5 expression is increased in mesenchymal stem cells from systemic lupus erythematosus (SLE) patients [32], BMP4 and BMP5 expression in synovial tissues from osteoarthritis and RA patients [33], and BMP2 and BMP6 expression in the arthritic synovium from RA and spondyloarthropathy patients [34] were decreased. Despite the intriguing expression data from a variety of autoimmune disorders, the immunological importance of BMPs in relation to these systemic autoimmune diseases has not been clearly demonstrated [1].

Mounting recent evidence suggests that activin A and BMPs have immunological roles, but only limited studies, especially in systemic autoimmune diseases, have been reported, in contrast to the TGF-βs [1]. Further investigation of TGF-β superfamily members in immune responses is needed in order to guide future strategies to develop therapies for systemic autoimmune diseases.

## 3. Expression of TGF-βs in Immune Cells

Immune cells are major sources of TGF-βs for regulating immune responses [35]. Since TGF-β1 is the predominantly expressed isoform in the immune system [5], the production and function of TGF-β1 has been preferentially investigated.

Conditional deletion of *Tgfb1* in T cells results in systemic autoimmune inflammation, demonstrating the fact that TGF-β1 produced by T cells helps maintain immune homeostasis [36]. Tregs are known to be one of the major producers of TGF-β1 [5,37]. Thymus-derived CD4^+^CD25^+^ Tregs produce high levels of TGF-β1 in a cell surface-bound form [38], and Tregs developed in the periphery, such as T regulatory 1 (Tr1) cells, secrete TGF-β1 [39,40]. Although FoxP3^+^ Treg-derived TGF-β1 could be redundant in Treg-mediated immune tolerance in some conditions [35,41], TGF-β plays a crucial role in Treg-cell-mediated suppression of T cells in vivo [42]. In other T cell subsets, activated C-X-C chemokine receptor type 5 (CXCR5) expressing follicular helper T (T_FH_) cells are reported to produce TGF-β1 [43]. CXCR5^+^ T cell subsets include both T_FH_ cells and recently identified follicular regulatory T (T_FR_) cells [44,45]; thus, further evaluation of the cellular source of TGF-β1 among CXCR5^+^ T cell subsets is required.

It is well known that lymphocytes other than T cells also produce TGF-β. Lipopolysaccharide (LPS)-activated B cells produce surface TGF-β1 that exerts inhibitory effects on CD8^+^ T cells [46], and neuroinflammation was exacerbated when the *Tgfb1* gene was conditionally deleted in B cells, highlighting the important regulatory roles of B cell-derived TGF-β1 [47]. TGF-β1 production from B cells is reduced upon co-engagement of the B cell receptor (BCR) and Toll-like receptor (TLR) 9, and the suppression of B cell-derived TGF-β1 with stimulation could lead to a breakdown in immune tolerance [48]. Another lymphocyte subset, NK cells, also produce TGF-β1 [49], which is important for anti-tumor NK cell activity [50].

Myeloid cells including macrophages, dendritic cells (DCs), mast cells, and eosinophils also produce TGF-β1. Myeloid cell-specific deletion of *Tgfb1* attenuated progression of heterotropic ossification induced by Achilles tendon puncture [51]. More specifically, LPS-stimulated macrophages produce TGF-β1, which induces B cells to secrete IgA antibody [52], and bone marrow-derived immature DCs rather than mature DCs produce TGF-β1 [53]. Mast cells are unique in that they co-secrete latent TGF-β1 and the activating enzyme chymase 1 that activates TGF-β1 [54]. In granulocytes, TGF-β1 derived from tissue-resident eosinophils in airway [55] and intestine [56] plays important roles for homeostasis in peripheral tissues [57]. Furthermore, recently identified innate lymphoid cells (ILCs) resident in the intestine produce TGF-β1, which expands regulatory ILCs during inflammation in an autocrine manner [58].

In contrast to TGF-β1, according to the literature currently available, the amount of TGF-β2 in the immune system is negligible, and the production of TGF-β3 from immune cells has only recently been recognized [9,10]. TGF-β3 mRNA is reported to be expressed in lymphocytes such as CD4^+^ T cells, CD8^+^ T cells, γδT cells, and B cells [10]. At the protein level, TGF-β3 is highly produced from CD4^+^CD25^−^LAG3^+^ Tregs (LAG3^+^ Tregs), with lesser amounts of TGF-β3 produced by Th1 cells and Th17 cells [59]. We have previously reported that LAG3^+^ Tregs suppress systemic humoral immune responses in a TGF-β3-dependent manner [59], and that TGF-β3 production from LAG3^+^ Tregs is significantly reduced when the expression of transcriptional factors early growth response gene 2 (Egr2) and Egr3 is deficient or if Fas is mutated [59,60]. Another group reported that Egr2-deficient mice develop lupus-like autoimmune disease [61]. We previously verified that polymorphisms in *EGR2* are associated with susceptibility to SLE [62], and that Egr2 is characteristically expressed in LAG3^+^ Tregs [63,64], which have the potential to ameliorate lupus pathology [59]. TGF-β3’s role as a regulatory molecule of LAG3^+^ Tregs and its inhibitory effect on systemic autoimmune diseases, including SLE, warrants further investigation.

## 4. Synthesis of TGF-β3

The biological activities of TGF-βs are regulated at multiple steps, including synthesis, proteolytic processing, secretion, and activation [65]. All three TGF-β isoforms are initially synthesized as an inactive pre-pro-TGF-β precursor, and the removal of the signal peptide, homo dimerization of pro-TGF-β, and subsequent cleavage by furin convertase forms a small latent complex in which the mature TGF-β ligand and its latency-associated peptide (LAP) are non-covalently connected [65]. The LAP renders the biological activities of TGF-βs latent by shielding the receptor-binding epitopes in the mature ligand [65,66]. Subsequently, the small latent complex covalently binds to latent TGF-β binding protein (LTBP) to form the large latent complex, which interacts with components of the extracellular matrix (ECM) [10,65]. 

**Figure 1:**
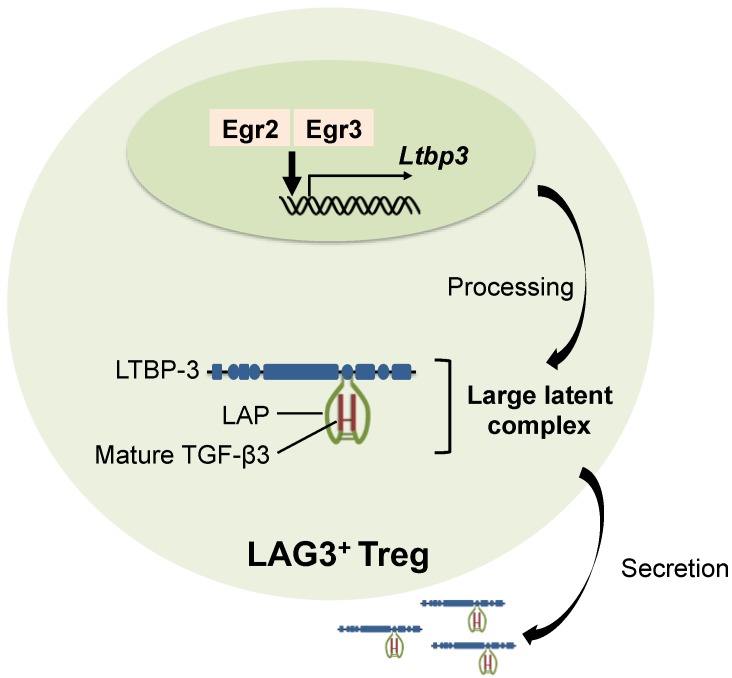
The roles of Egr in TGF-β3 production in CD4^+^CD25^−^LAG3^+^ regulatory T cells. Synthesized latency-associated peptide (LAP) and mature TGF-β3 binds to latent TGF-β binding protein-3 (LTBP-3), and the large latent complex is secreted from cells. Early growth response gene 2 (Egr2) and Egr3 regulate the expression of Ltbp3, which leads to efficient secretion of TGF-β3 from CD4^+^CD25^−^LAG3^+^ regulatory T cells (LAG3^+^ Tregs).

LTBPs are multi-domain glycoproteins that interact with fibrillin microfibrils [67]. Of the four isoforms of LTBP, LTBP-1 and LTBP-3 bind to all three TGF-β isoforms, whereas LTBP-2 does not bind the LAP of any TGF-β isoform, and LTBP-4 is supposed to interact only with the TGF-β1 LAP [68]. The disulfide bond between LAP and LTBP is an bridge between cysteine residues in the LAP and LTBP [69]. Genetically mutated mice with the cysteine residue in the LAP changed to serine exhibit systemic inflammation, although not as strong as *Tgfb1* knockout mice, and reduced serum TGF-β1 levels; thus, the association of latent TGF-β1 with LTBP is important for extracellular TGF-β1 activity [70]. However, the specific binding of LTBP-1 or LTBP-3 to each TGF-β isoform has not been clearly demonstrated [71]. Recently, we reported that secretion of TGF-β3 by LAG3^+^ Tregs is regulated by LTBP-3 in T cells (Figure 1) in a murine model of lupus [60]. Additionally, deletion of Egr2 and/or Egr3 decreases Ltbp3 expression [60]; thus Egr2/Egr3 could be critical for TGF-β3 synthesis. Further investigation of the role of LTBPs in the secretory mechanics of TGF-β would lead to insightful findings in immune responses.

Activation of latent complexes of TGF-βs is essential in order to exert their biological functions [35,42]. TGF-β1 and TGF-β3 have an integrin-binding arginylglycylaspartic acid (RGD) motif, which is recognized by α_v_ integrins and is required for integrin-mediated activation [71,72]. One groups showed that genetically modified mice harboring a nonfunctional variant of the RGD sequence in the *Tgfb1* gene develop fatal multiorgan inflammation identical to *Tgfb1* knockout mice [73]. Among the members of the integrin receptor family, integrin α_v_β_6_ and α_v_β_8_ have the capacity to bind and activate pro-TGF-β1 and pro-TGF-β3 [74], and the combined genetic loss of α_v_β_6_ and α_v_β_8_ integrins reproduces the phenotypes of *Tgfb1* and *Tgfb3* knockout mice [75]. Conditional deletion of the *Igtb8* gene encoding integrin β8 in leukocytes results in systemic inflammation with autoantibodies [76]. Further, deficiency of integrin α_v_β_8_ in Tregs, which display less active TGF-β levels, abrogates their suppressive abilities on T cell responses [42]. The elaborate machinery of TGF-β synthesis is carefully regulated by the immune system, and the recent findings of TGF-β isoform-specific function suggest the importance of investigating further TGF-β isoform-specific synthesis mechanisms. 

## 5. TGF-β3 Signal Transduction

TGF-βs signal through a hetero-tetrameric receptor complex composed of two type I receptors and two type II receptors [77]. The constitutively active cytoplasmic domain of TGF-β type II receptors (TβRII) phosphorylates type I receptors on serine and threonine residues in response to the binding of mature TGF-βs, after which the activated type I receptors canonically phosphorylate Smad proteins [77,78]. TβRII interacts with TGF-β type I receptors (TβRI), also called activin receptor like kinase (ALK)-5, which typically induces the phosphorylation of Smad2 and Smad3, and also interacts with ALK-1, which typically induces the phosphorylation of Smad1 and Smad5 [79,80]. In endothelial cells, ALK1 antagonizes ALK5-mediated Smad2/Smad3 signaling, and the ratio of ALK5 to ALK1 determines the responsiveness to TGF-βs [79,81]. Further, type III receptors, such as endoglin and betaglycan, regulate the access of TGF-βs to the type I and type II receptors [82]. Although TGF-β1 and TGF-β3 can interact with type II receptors even without type I receptors, TGF-β2 interacts very weakly with type II receptors, and type I or type III receptors are required for binding [80,82]. In association with TβRII, TGF-β1 and TGF-β3, but not TGF-β2, interact with endoglin [82,83].

Like TGF-β1 [84], the initial report demonstrated that TGF-β3 dominantly drives Smad2-dependent ALK-5 signaling in palatal fusion [85]. During palatogenesis, conditional deletion of *Trim33* and *Smad4* in epithelium in concert with TGF-β activated kinase-1 (Tak1) inhibition phenocopied the palate defects observed in epithelium-specific *Tgfb3*-deficient mice; thus TGF-β3 is supposed to transduce signals via both canonical Smad-dependent and non-canonical Smad-independent signaling [86]. In human B cells, we recently found that TGF-β3 induces phosphorylation of Smad1/5 along with Smad2 and Smad3 [87], as previously reported in TGF-β1-treated B cells [88]. The differential signal transduction observed for TGF-β1 and TGF-β3 is linked to different downstream functions, at least for CD4^+^ T cells. TGF-β3 produces highly pathogenic Th17 cells and induces more phosphorylation of Smad1 and Smad5, but less Smad2 and Smad3 phosphorylation, than TGF-β1 [89], although further studies of the different downstream signaling pathways of TGF-β3 and TGF-β1 are warranted.

## 6. TGF-β3 in Immune Responses

TGF-βs are pluripotent cytokines with both pro-inflammatory and anti-inflammatory effects, depending upon specific immune contexts such as cellular targets, cytokine concentrations, tissue microenvironments, and cytokine synergy [9,10,77,90]. In contrast to the previous reports describing a variety of immunological roles for TGF-β1, a direct immunological role for TGF-β3 has not been described in detail until recently. Although *Tgfb1*-knockout mice develop fatal systemic autoimmune inflammation in heart, lungs, pancreas, colon and salivary glands [6,7], the immunological abnormalities in *Tgfb3*-knockout mice have not been reported due to death shortly after birth due to cleft palate formation and impaired lung development in *Tgfb3*-knockout mice [91,92].

### 6.1. Pro-Inflammatory Roles of TGF-β3

Similar to TGF-β1 [5,9,37], TGF-β3 has pro-inflammatory effects. Derepression of TGF-βs, especially Tgfb3 mRNA, results in the accumulation of Th17 cells and Foxp3^+^ T cells in T cell-specific deletion of tripartite motif protein 28 (Trim28) [93]. Further analyses indicate an isoform-specific function of TGF-βs in Th17 cells in that a combination of TGF-β3 and IL-6 induces highly pathogenic Th17 cells, compared to the Th17 cells induced by TGF-β1 plus IL-6 [89].

In B cells, low concentrations of TGF-β1 enhance T cell-dependent sheep erythrocyte-induced B cell proliferation [94]. In addition, TGF-β1 enhances IgG2b and IgA production from LPS-induced B cells [95]. A direct pro-inflammatory role of TGF-β3 has not been reported, but our latest research indicates that antibody production in LPS-stimulated B cells is enhanced with TGF-β3 [96]. TGF-β concentration is one of the determinants for its pro-inflammatory effects. TGF-β1 shows concentration-dependent bifunctional effects in B cells [94], and we have verified that TGF-β3 also has concentration-dependent bifunctional effects [87,96]. Although a combination of TGF-β1 and IL-21 generates mucosal-homing IgA-secreting plasmablasts [43], a role for TGF-β3 in mucosal immunity has not been demonstrated. Since TGF-βs show context-dependent pro-inflammatory effects on B cells, different concentrations and a combination of cytokines should be utilized when investigating their immunoregulatory roles.

### 6.2. Anti-Inflammatory Roles of TGF-β3

Unlike TGF-β1 [5,37], direct anti-inflammatory roles for TGF-β3 in vivo had not been clearly documented until our recent reports. Targeted recombination of the coding sequence of mature TGF-β1 with a sequence from TGF-β3 partially prevented autoimmune diseases caused by TGF-β1 deficiency, indicating that TGF-β3 is not fully interchangeable with TGF-β1 and that they have distinct immunoregulatory roles [97]. In T cells, TGF-β3 inhibits the differentiation of Foxp3-expressing CD4^+^ T cells [98]. Moreover, treatment with a TGF-β3 neutralizing antibody prevented TGF-β3-expressing LAG3^+^ Treg-mediated suppression [59]. Although TGF-β1 is known to inhibit the differentiation of Th1 [99] and Th2 cells [100], and suppress the activation of NK cells [101] and macrophages [102], the effects of TGF-β3 on these cell subsets has not yet been described.

B cell-specific deletion of TβRII results in B cell hyperresponsiveness and virtually complete serum IgA deficiency, indicating that TGF-βs could have a regulatory role in T cell-independent B cell homeostasis [103]. TGF-β1 inhibits anti-IgM-simulated B cells by suppressing the phosphorylation of spleen tyrosine kinase (Syk), and inhibits IL-4-stimulated B cells by suppressing the phosphorylation of signal transducer and activator of transcription-6 (STAT6) [104]. Similar to TGF-β1, TGF-β3 inhibits B cell-proliferation and antibody production stimulated by anti-IgM, IL-4, or anti-CD40 plus IL-4 by suppressing the phosphorylation of Syk, STAT6, or NF-κB p65, respectively [59]. High-affinity antibodies are generated through the interaction of T_FH_ cells with germinal center B (GCB) cells [105,106], and TGF-β3, as well as TGF-β1, suppresses GCB cells in vitro [60]. Lastly, systemic administration of TGF-β3-expressing vectors, but not TGF-β1-expressing vectors, inhibited T cell-dependent humoral immune responses by suppressing the development of GCB cells [96].

**Figure 2:**
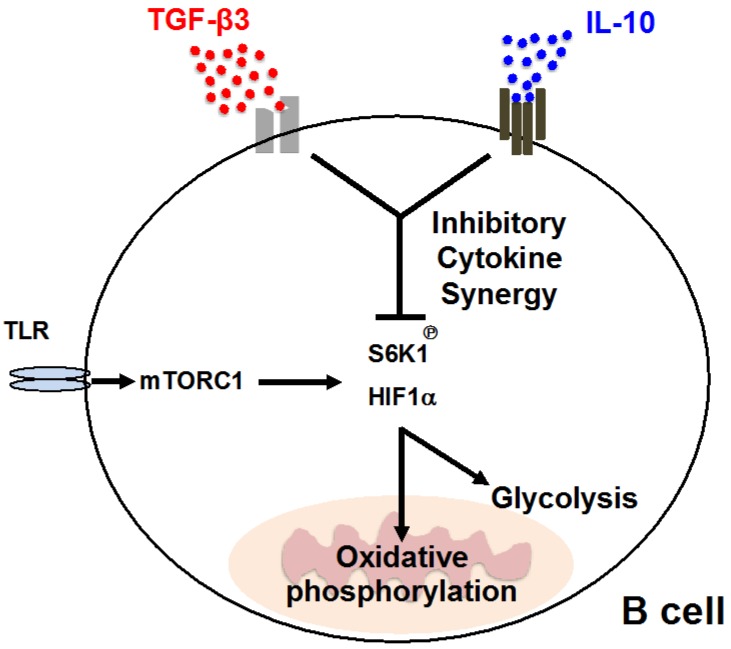
Schematic description of the “inhibitory cytokine synergy” of TGF-β3 and IL-10 in activated B cells. Phosphorylation of S6 kinase 1 (S6K1) and expression of hypoxia inducible factor 1α (HIF1α), which is a downstream signaling of mammalian target of rapamycin complex 1 (mTORC1), are suppressed by a combination of the inhibitory cytokines TGF-β3 and IL-10. ICS further suppresses B cell cellular energetic metabolism, such as glycolysis and oxidative phosphorylation. Arrows, positive regulation; T-bar, negative regulation.

In contrast, both TGF-β1 and TGF-β3 enhance antibody production by LPS-stimulated B cells. We recently reported that TGF-βs exhibit inhibitory effects against LPS-stimulated B cells in the presence of IL-10 [96], which is a comprehensive inhibitory cytokines [107]. Cytokine synergy is a phenomenon of greater effects than the sum of its parts [108], and our report is the first to propose inhibitory cytokine synergy (ICS) [96], which is defined as a coordinated inhibitory ability of two or more cytokines, but not either cytokine alone. The ICS effects of TGF-β3 and IL-10 regulate TLR-mediated humoral immune responses both in vitro and in vivo by suppressing mammalian target of rapamycin (mTOR) signaling. The inhibition of mTOR signaling in B cells by ICS effects further suppresses cellular metabolism, including glycolysis and mitochondrial oxidative phosphorylation (Figure 2). TGF-β3 ameliorates IL-10-sufficient lupus-prone MRL/*Fas^lpr/lpr^* (MRL/*lpr*) mice [59], but the requirement of both TGF-β3 and IL-10 in the regulation of other lupus models induced with a TLR7 agonist [109] implies the importance of considering ICS when designing future therapeutic strategies. Irrespective of the presence of IL-10, high concentrations of TGF-β3, like TGF-β1, inhibit proliferation, antibody production, and the differentiation of human B cells activated with various stimuli [87]. However, a physiological concentration of TGF-β3 enhances activated human B cells, which notably requires the presence of IL-10 [96]. Collectively, TGF-β3 has the potential to regulate systemic autoimmune diseases by inhibiting B cells. Context-dependent functionality of TGF-β3, like TGF-β1, could be an obstacle for clinical application and adequate inhibitory conditions in immune responses should be further investigated. 

## 7. TGF-β3 and Human Diseases

Recent advances in understanding the biological functions of TGF-β3 are incrementally uncovering the roles of TGF-β3 in human diseases. As mentioned above, Tgfb3 knockout mice die perinatally, and immunological abnormalities have not been reported [91,92]. In humans, *TGFB3* mutations are associated with cardiovascular involvement, including aortic aneurysm, dissection, mitral valve diseases, and systemic features overlapping with Loeys–Dietz, Shprintzen–Goldberd and Marfan syndromes [110]. Although several family members with autoimmune features, including HLA-B27-positive spondyloarthritis, Graves’s disease, and celiac disease [110] have been identified, a linkage between *TGFB3* mutations and autoimmunity has not been studied in detail.

TGF-βs are thought to be central mediators of systemic sclerosis (SSc) pathogenesis, because of the pleiotropic effects of TGF-βs in fibrosis, inflammation, and vascular biology [111]. Immunohistochemical analyses indicate that the expression of all three isoforms of TGF-βs are increased in skin from SSc patients [111]. In addition, the expression of TGF-β-regulated genes, such as cartilage oligomeric matrix protein (COMP) and collagen type V α2 (COL5A2), is upregulated in lungs from SSc patients [112]. Although no significant differences in total serum TGF-β1 levels between SSc patients and healthy controls have been observed, serum-active TGF-β1 levels in diffuse cutaneous SSc patients are negatively correlated with skin score and lower than active TGF-β1 levels in limited cutaneous SSc patients and healthy controls [113]. In animal models with blocked TGF-β signaling, inhibition of ALK5 attenuates bleomycin-induced pulmonary fibrosis [114], and LAP prevents sclerodermatous graft-versus-host disease [115]. Metelimumab, which is a human monoclonal antibody that specifically targets TGF-β1, failed in phase I/II trials for SSc [116]. In contrast, fresolimumab, which is specific for TGF-β1 with high affinity in addition to TGF-β2 and TGF-β3, benefited SSc patients, and further studies for the safety and longer-term use effects are expected [117]. A genome-wide association study (GWAS) of SSc patients in African Americans recently identified *TGFB3* as a SSc susceptibility gene [118]. These results collectively suggest that TGF-β3 could be an important mediator and drug target for SSc.

In SLE, the roles of TGF-βs are expected to be protective. For examples, TGF-β1 ameliorates murine lupus [119,120], and serum TGF-β1 concentrations are low in active SLE patients [121,122]. Additionally, an impaired response to a TGF-β1-mediated anti-proliferative effect against peripheral blood mononuclear cells from active SLE patients has been reported [123]. In relation to TGF-β3, mice with Egr2/Egr3 deficiency, which leads to the reduction of TGF-β3 secretion from LAG3^+^ Tregs, develop lupus-like diseases [60]. Furthermore, therapeutic effects of TGF-β3 in murine lupus [59,96], and the reduction of TGF-β3-secreting LAG3^+^ Tregs in SLE patients [59] imply potential preventive roles of TGF-β3 in SLE. 

TGF-β1 and TGF-β3, in combination with other cytokines, regulate both encephalitogenic T cells and regulatory T cells [9,89], although precise roles for TGF-βs in MS have not been elucidated. Mice with T cell-specific deletion of *Tgfb1* are resistant to EAE and exhibit less Th17 cell differentiation [41], although the in vivo roles of TGF-β3 in a mouse model of EAE have not been reported. Since CD4^+^ T cells from MS patients show reduced levels of TGF-β signaling components [124], the initial failure of a clinical trial using TGF-β2 for MS [125] could be due to the lowered responsiveness of TGF-βs in the pathologies of MS. Further evaluations of TGF-β signaling in MS that incorporates recent understandings of TGF-β3’s impact on pathogenic Th17 cells are to be expected [126].

Targeting TGF-β pathways as a therapeutic strategy has been clinically applied outside the field of immunology. TGF-β is considered to be a critical regulator of fibrosis; TGF-β1 and TGF-β2 have pro-fibrotic effects and promote fibroplasia, whereas TGF-β3 exerts anti-fibrotic effects and reduces scar formation [9]. A humanized monoclonal antibody directed against integrin α_v_β_6_, which activates pro-TGF-β1 and pro-TGF-β3 [74], is currently in phase II trials in patients with idiopathic pulmonary fibrosis [127]. Although human recombinant TGF-β3, avotermin, failed in phase III clinical trials [128], avotermin improved wound healing without serious adverse events in phase I/II clinical trials for a prophylactic anti-scarring therapy [129]. In the field of oncology, regulation of TGF-β activity in tumor microenvironments and targeting TGF-β signaling as a novel immunotherapy has been in the news [130,131]. The TβRI-specific inhibitors galunisertib and fresolimumab, in combination with other drugs, have been in clinical trials for a variety of tumors [130]. The systemic effects, immunological changes, and safety with these novel medications that modulate TGF-β signaling provide insightful information for future therapeutic strategies for systemic autoimmune diseases. 

## 8. Concluding Remarks

The pluripotent TGF-β cytokines have been investigated in a variety of fields. Although the three isoforms of TGF-β each have distinct biological roles, TGF-β1 has been paid particular attention regarding its ability to regulate immunity. Recent identification of pro- and anti-inflammatory roles for TGF-β3 [9,10] highlights the importance of the evaluation of TGF-β isoform-specific immunological roles. Since TGF-β3—like TGF-β1—has a context-dependent functionality in immunity [90,96], considering concentrations and combination with other cytokines is important when evaluating the pathogenic and pathoprotective roles of TGF-β3. Investigations of TGF-β3 by conditionally mutated mouse models, together with evaluations of the physiological and pathological roles of TGF-β3 in humans, would be helpful for further progress in assessing TGF-β3-mediated immune responses.
